# Advances in Molecular Research of Tropical Fruit

**DOI:** 10.3390/ijms252413582

**Published:** 2024-12-19

**Authors:** Yonghua Qin

**Affiliations:** Guangdong Provincial Key Laboratory of Postharvest Science of Fruits and Vegetables and Key Laboratory of Biology and Genetic Improvement of Horticultural Crops (South China), Ministry of Agriculture and Rural Affairs, College of Horticulture, South China Agricultural University, Guangzhou 510642, China; qinyh@scau.edu.cn

Fruit trees, similar to other edible plants, hold immense commercial value within the agricultural sector [1,2]. Beyond their economic importance, many fruit trees provide notable medicinal and health benefits. They contribute to human well-being by offering essential resources in terms of their fruits, roots, stems, leaves, flowers, and seeds. Fruit crops play a critical role in global food production systems and are key to maintaining food and nutritional security [3,4]. Given the rapidly growing global population and evolving dietary preferences, there is an increasing need to sustainably boost fruit and nut production to ensure long-term food security [5]. Tropical fruits are of immense agricultural and economic importance worldwide [6]. The cultivation of these fruits faces numerous challenges, including environmental stresses, vulnerability to pests and diseases, and the demands for improved quality traits such as flavor, nutritional content, and shelf life [7]. The application of molecular biology and genomics has the potential to transform the way tropical fruits are cultivated, providing tools to overcome these challenges [8,9,10].

Tropical fruits are cultivated in warm, humid regions near the Equator, extending north to the Tropic of Cancer and south to the Tropic of Capricorn. There are over 300 tropical fruit varieties that yield edible produce, including popular fruits such as bananas, pineapples, lychees, longans, mangoes, pitaya, passion fruits, papayas, rambutans, açaí, jackfruits, mangosteens, avocados, guavas, durians, cherimoyas, cashews, and macadamia nuts [11]. Many developing countries rely on tropical fruits as a key source of export revenue and income, while also benefiting from their high nutritional values. The cultivation and production of tropical fruits are expanding on a global scale [12]. Tropical fruits are highly perishable and tend to be especially sensitive to environmental factors, as well as biotic and abiotic stresses [13]. This Special Issue, “Advances in Molecular Research of Tropical Fruit”, brings together innovative research focusing on the genetic and molecular mechanisms that govern key traits in tropical fruits. The articles explore a wide range of approaches, from genomics and transcriptomics to metabolomics and gene editing, all aimed at advancing the understanding and improvement of tropical fruit species. In this review, we summarize the findings from all nine contributions to this Special Issue, highlighting how these advances are shaping the future of tropical fruit research and cultivation.

The manuscript “Advancements in Reference Gene Selection for Fruit Trees: A Comprehensive Review” presents an in-depth analysis of the pivotal role of reference genes in ensuring accurate gene expression measurements in fruit tree research, particularly through real-time quantitative polymerase chain reaction (qRT-PCR) [14]. As qRT-PCR has become a cornerstone in molecular biology for its precision, sensitivity, and throughput, the need for stable reference genes, which normalize gene expression data, has become increasingly significant [15,16,17,18]. The review thoroughly discusses the challenges, advancements, and future directions in the selection of reference genes, which are essential for minimizing variations in RNA quantity, quality, and reverse transcription efficiency across different samples and experimental conditions [3]. No universal reference gene can be applied across all tissues, species, or experimental conditions. The expression stability of reference genes varies considerably depending on factors such as the species, organ type, and physiological or environmental conditions [19]. For instance, commonly used reference genes such as *ACT*, *GAPDH*, and *EF-1α* have been shown to exhibit instability under certain experimental setups, necessitating the need for rigorous validation before their application in qRT-PCR studies [20].

The manuscript reviews the evolution of methods for evaluating reference gene stability in molecular biology. Initially, simple tools were used, but modern approaches now incorporate advanced algorithms [21,22] such as BestKeeper, geNorm, and NormFinder, which analyze statistical parameters to identify stable reference genes. These tools improve the reliability of gene selection, particularly when multiple genes are needed for accurate normalization [1,23]. The review highlights advancements in fruit trees, such as *18S rRNA* and *ACTB* in citrus [24], *GAPDH* and *gyrβ* in mango [25,26], and *EF-1α* and *Mn-SOD* in longan [27]. The manuscript also emphasizes the potential of RNA-Seq and machine learning for identifying novel, stable reference genes across various conditions [28]. It advocates for the continued validation of reference genes in specific stress contexts and calls for integrating new technologies to enhance the accuracy and reproducibility of qRT-PCR analyses in fruit tree research. In conclusion, this comprehensive review provides an invaluable resource for researchers in molecular biology, particularly those focusing on fruit trees, by addressing both current practices and emerging trends in reference gene selection. The findings underscore the importance of context-specific validation and advocate for the adoption of cutting-edge technologies to ensure the precision of future qRT-PCR analyses in fruit tree research [29].

The review by Shah et al., titled “Pitaya Nutrition, Biology, and Biotechnology”, provides a comprehensive analysis of pitaya, emphasizing its nutritional, biological, and biotechnological importance [30]. Native to Central and South America, pitaya has gained attention for its rich nutritional profile and adaptability to tropical and subtropical climates. The review discusses advancements in taxonomy, propagation, and genetic improvement, highlighting biotechnology’s role in enhancing the crop. Pitaya is noted for its high levels of bioactive compounds, including betalains, phenolic acids, flavonoids, vitamins (B1, B2, B3, C), and essential minerals such as potassium, calcium, and magnesium. Red-fleshed pitaya varieties are particularly valued for their strong antioxidant capacities, driven by betalains and phenolic compounds [30]. Comparative analyses of *H. undatus*, *H. polyrhizus*, and *H. megalanthus* reveal significant variations in nutrient content, making pitaya an important source of natural antioxidants and anti-inflammatory agents [31]. These compounds have been linked to benefits such as anti-cancer, anti-diabetic, and cardiovascular protection. Betalains, hydrophilic nitrogen-containing pigments found in pitaya, serve as powerful antioxidants, protecting against oxidative stress, inflammation, and certain cancers [30]. The review details the key enzymes involved in betalain biosynthesis, such as tyrosinase and 4,5-DOPA-extradiol-dioxygenase, which regulate the production of betalain pigments, specifically betacyanins and betaxanthins [19].

Recent genetic research on transcription factors (TFs) (HmoWRKY42, HuMYB132, and HubHLH159) have illuminated betalain biosynthesis control, enabling biotechnological increase of pitaya color production [30]. Pitaya has antioxidant, anticancer, and antimicrobial properties [32]. Due to their high phenolic and flavonoid contents, fruit peel and pulp reduce oxidative stress and the risk of chronic illnesses including cancer and cardiovascular disease [30]. Pitaya is also known to have antibacterial properties against pathogens such as *Staphylococcus aureus* and *Escherichia coli*, making it a promising natural source for nutraceuticals and therapeutics [33]. Tissue culture, micropropagation, and molecular markers have improved pitaya’s disease resistance and fruit quality [19,34]. Molecular marker technologies including SSR and AFLP have also helped genetic diversity research and germplasm conservation, assuring sustainable agricultural development [30,35]. Despite significant advancements, challenges such as vulnerability to pests, and diseases like *Neoscytalidium* stem canker persist [36]. The study recommends using CRISPR-Cas9 to increase fruit quality and disease resistance. Breeding efforts with high-throughput sequencing and genomic selection may produce better pitaya cultivars [30].

Pitaya is a nutritious fruit with intriguing biotechnological uses. Tissue culture, molecular biology, and genetic engineering will boost pitaya output, stress tolerance, and nutritional value. With continuous studies, pitaya has become an important tropical and subtropical fruit crop, solving agricultural and health issues.

The manuscript by Lin et al. (2023) investigates the molecular and metabolic mechanisms behind ethephon-induced flowering in pineapple [37]. Flowering in pineapple is a critical process that requires precise control to optimize yield and synchronize harvests [38]. Ethephon, an ethylene-releasing compound, is used to promote flowering and synchronize harvests, but the underlying pathways were unclear. Through metabolomic and transcriptomic analyses, the study identified 804 metabolites and thousands of differentially expressed genes (DEGs), particularly in carbohydrate metabolism and hormone signaling [37]. Ethylene is known to promote pineapple flowering by reducing auxin levels [39] and this study confirmed that a significant decrease in indole-3-acetic acid (IAA) occurred during flower induction, underscoring its regulatory role [37]. Changes in sugars (e.g., sucrose, stachyose) and hormones like indole and gibberellins (GA) were analyzed. Ethephon reduced GA and IAA, crucial for flower induction, and increased the expression of genes involved in sugar metabolism, such as *Sucrose Synthase 2* (*SS2*) and *Trehalose Phosphate Synthase 9* (*TPS9*). Important genes, including *AGL9*, *ETIL3*, and *AP2*, were linked to floral development and ethylene signaling. By integrating the data, the researchers constructed a regulatory network of 48 genes and 32 metabolites, revealing complex interactions between hormones, sugars, and TFs [40]. By integrating metabolomic and transcriptomic analyses, Lin et al. (2023) uncovered key metabolites and genes involved in sugar metabolism and hormone signaling, highlighting their roles in flower induction. The identified regulatory network provides a foundation for future research aimed at enhancing pineapple flowering control and yield optimization through biotechnological interventions [37].

A study by Cao et al. (2024) explores the physiological, metabolic, and transcriptomic mechanisms underlying the proliferation and somatic embryogenesis (SE) of *Litchi chinensis* embryogenic callus (EC) promoted by D-arginine (D-Arg) treatment [41]. The research provides crucial insights into how D-Arg facilitates EC proliferation and SE, focusing on polyamine metabolism, endogenous hormones, and gene expression changes. Polyamines (PAs) such as putrescine (Put), spermidine (Spd), and spermine (Spm) play critical roles in plant development, especially in embryogenesis and organogenesis [42]. D-Arg was found to significantly alter PA metabolism by inhibiting the activity of diamine oxidase (DAO) and polyamine oxidase (PAO), reducing hydrogen peroxide (H_2_O_2_) production, and promoting EC proliferation [41]. The reduction in Put content and the increased (Spd + Spm)/Put ratio following D-Arg treatment were linked to enhanced somatic embryo induction, as higher ratios of Spd and Spm to Put are crucial for SE in various plant species. The study further showed that the application of exogenous D-Arg inhibited arginine decarboxylase (ADC) activity, a key enzyme in Put biosynthesis. This led to a significant reduction in Put levels, while Spd and Spm levels remained elevated during the later stages of SE. These changes in PA metabolism are key factors in D-Arg-induced SE, as they regulate cell division, differentiation, and the formation of somatic embryos [41]. D-Arg also influenced endogenous hormone levels in litchi EC [43], playing a pivotal role in the regulation of SE. The levels of auxins (IAA-Gly), cytokinins (K9G and DHZ7G), and jasmonic acids (JA, JA-Val, and JA-ILE) were significantly altered by D-Arg treatment [41]. The increase in IAA-Gly and cytokinins, coupled with a decrease in JA levels [44], is consistent with previous findings that auxins and cytokinins promote SE by stimulating cell division and differentiation, while JAs tend to inhibit embryogenesis. Moreover, a proper hormone balance is essential for SE. Elevated levels of *IAA-Gly* and *DHZ7G* promote early embryo formation, while reduced JA levels remove inhibition, facilitating the callus-to-embryo transition. Key TFs such as WRKY, AP2/ERF, and C_2_H_2_ were identified, supporting SE through stress regulation and hormone signaling. KEGG analysis showed pathways related to cell wall biogenesis and protein synthesis, demonstrating D-Arg’s role in enhancing callus proliferation and embryo development [41]. This study provides valuable insights into the molecular mechanisms by which D-Arg promotes EC proliferation and SE in *Litchi chinensis*. The regulation of PA metabolism, hormonal balance, and gene expression all play pivotal roles in this process. Future research may explore the potential application of D-Arg in other plant species to optimize SE protocols, as well as investigate its broader role in plant development and stress tolerance.

A study by Yu et al. (2023) presents a comprehensive analysis of post-transcriptional regulatory mechanisms, specifically alternative splicing (AS) and alternative polyadenylation (APA), during watermelon (*Citrullus lanatus*) fruit ripening [45]. Using PacBio single-molecule long-read sequencing, this research uncovers the complexity of transcriptomic regulation in fruit ripening, a process vital for traits such as texture, flavor, and sugar content. While transcriptional regulation has been well studied, the roles of AS and APA in fruit ripening remain underexplored. AS creates multiple transcript isoforms from one gene, enhancing protein diversity and functional regulation. APA alters the 3′ untranslated regions (UTRs) of mRNAs, influencing mRNA stability, localization, and translation efficiency, adding an additional layer of post-transcriptional regulation [45]. Watermelon fruit ripening was accompanied by extensive AS events [46,47,48]. The researchers identified 42,285 splice isoforms from over 6.9 million full-length reads, with 16,495 AS events, primarily intron retention (IR), followed by exon skipping (SE) and alternative 5′ splice sites (A5). AS events were enriched in metabolic pathways such as fructose, mannose, starch, and carotenoid metabolism, all essential for sugar content and pigmentation during ripening. Notably, the PDS gene, crucial for carotenoid biosynthesis, showed IR during ripening, confirming AS’s role in carotenoid metabolism [49]. The APA analysis identified 21,506 polyadenylation sites across 11,611 genes, with key ripening-related genes including *Sucrose Synthase* (*SUS*) and *Phytoene Synthase* (*PSY*) influenced by APA, affecting sugar accumulation and pigment synthesis. APA events altered mRNA transcript lengths, impacting gene expression, and genes with APA sites had longer introns, suggesting they help produce shorter transcripts. Additionally, 594 lncRNAs were identified, showing stage-specific expression and regulating key ripening genes. Overall, the study demonstrates the importance of AS, APA, and lncRNAs in controlling key metabolic pathways that determine fruit quality traits, such as sweetness and color, during ripening [45]. Future research could focus on validating these splice variants and APA events to further understand their roles in fruit biology.

Xu et al. (2023) explore the role of the *EjFAD8* gene in enhancing low-temperature tolerance in loquat (*Eriobotrya japonica*) [50], a subtropical fruit tree whose growth and development are vulnerable to cold stress due to its autumn–winter flowering and fruiting period [51,52]. Using transcriptomic and lipidomic approaches, *EjFAD8* is identified as a key fatty acid desaturase gene involved in the desaturation of membrane lipids, particularly sulfoquinovosyl diacylglycerol (SQDG) under cold conditions [52,53]. One of the primary mechanisms by which plants tolerate low temperatures is through the modification of membrane lipid composition [54]. Membrane lipids play a critical role in maintaining cell structure and function, and their unsaturation levels influence membrane fluidity and cold stress resistance [54,55]. Xu et al. demonstrated that the *EjFAD8* gene, encoding a fatty acid desaturase, plays a crucial role in cold tolerance by increasing the unsaturation of membrane lipids, particularly SQDG, to stabilize chloroplast membranes during cold stress and maintain photosynthetic efficiency. Overexpressing *EjFAD8* in *Arabidopsis thaliana* improved cold tolerance, resulting in less leaf wilting, greater root growth, reduced oxidative damage, and better membrane integrity, evidenced by reduced ion leakage and reactive oxygen species (ROS) accumulation. The study also linked *EjFAD8* to the *ICE-CBF-COR* signaling pathway, where its overexpression upregulated key *COR* genes such as *CBF1*, *CBF2*, *CBF3*, and downstream *COR* genes. This highlights a dual mechanism for enhanced cold tolerance: modifying membrane lipid composition and activating *COR* genes. The findings provide insights into loquat’s cold adaptation and offer a basis for breeding cold-tolerant subtropical crops [50].

The comprehensive study “Integration of Metabolomics and Transcriptomics to Explore Dynamic Alterations in Fruit Color and Quality in ‘Comte de Paris’ Pineapples during Ripening Processes” by Song et al. (2023) provides an in-depth exploration of the metabolic and transcriptomic changes that occur during the ripening of “Comte de Paris” pineapples (*Ananas comosus*) [56]. Pineapple ripening is a complex physiological process that involves significant transformations in fruit color, flavor, and texture factors that are critical to consumer acceptance and market value [57,58,59]. The authors used an integrative metabolomic and transcriptomic approach to investigate the molecular changes during pineapple ripening. They found that flavonoids and phenylpropanoids significantly contribute to fruit color, with anthocyanins and carotenoids driving pigmentation. Organic acids and lignin-related compounds influenced texture and flavor, balancing sweetness and acidity as the fruit matured. Key genes such as *FLS*, *FNS*, and *DFR* were identified as regulators of flavonoid biosynthesis, while *COMT*, *CCR*, and *CAD* were linked to texture modification through lignin metabolism [56].

The study provides valuable insights for breeding programs aimed at enhancing pineapple quality, focusing on traits such as color, texture, and flavor to improve postharvest quality and consumer satisfaction.

Banana (*Musa acuminata*) is one of the most widely consumed fruits globally, and its quality during ripening is a key determinant of consumer preference [60,61,62]. The ripening process involves complex biochemical changes, with starch degradation playing a central role in determining sweetness, texture, and overall fruit quality [63,64,65]. Alpha-amylase enzymes are pivotal in this conversion of starch into soluble sugars, which enhances the fruit’s appeal as it ripens [66]. However, despite the importance of starch metabolism in bananas, the molecular mechanisms underlying this process remain largely unexplored. The study by Sun et al. (2024) provides an in-depth analysis of the α-amylase gene family in bananas, with a focus on *AMY11* and its role in starch degradation during ripening [67]. Through genome-wide analysis, the authors identified 12 AMY proteins, classifying them into two groups, with *AMY11* showing the highest expression during mid-to-late ripening. Functional experiments confirmed that *AMY11* regulates starch breakdown—its silencing increased starch accumulation, while overexpression accelerated starch degradation, reducing amylose and amylopectin levels. *AMY11* was predominantly active in ripening fruit, and its localization in the chloroplast linked it to starch metabolism and photosynthesis. Phylogenetic analysis indicated its evolutionary conservation across monocots such as maize and rice [67]. This research highlights *AMY11* as a crucial regulator of banana ripening, with potential applications in improving postharvest quality traits including sweetness, texture, and shelf life through genetic engineering. Future studies could explore regulatory networks controlling *AMY11* expression to further enhance fruit quality.

Ascorbic acid (AsA), commonly known as vitamin C, plays a crucial role in plant defense, stress tolerance, and human nutrition due to its powerful antioxidant properties [68,69,70]. It is a key component of the ascorbate–glutathione cycle, helping to neutralize ROS and regenerate other antioxidants within plant cells [71]. While the biosynthesis and degradation of AsA have been widely studied, the molecular mechanisms regulating its accumulation during fruit development remain less understood. One of the key enzymes involved in this cycle is monodehydroascorbate reductase (MDHAR), which helps to regenerate AsA from its oxidized form, monodehydroascorbate [72,73]. However, its exact role in AsA regulation, particularly in fruit tissues, is still being explored. The study titled “Kiwifruit Monodehydroascorbate Reductase 3 Gene Negatively Regulates the Accumulation of Ascorbic Acid in Fruit of Transgenic Tomato Plants” explores the role of the *MDHAR3* gene from kiwifruit and its impact on AsA levels in transgenic tomato plants. MDHAR is a key enzyme in the recycling of AsA, which is vital for plant health and human nutrition. Among the seven *MDHAR* genes identified in kiwifruit, *AeMDHAR3* showed declining expression as the fruit matured. When *AeMDHAR3* was overexpressed in tomatoes, the activity of the MDHAR enzyme increased, but unexpectedly, AsA levels decreased. This was linked to an increase in ascorbate peroxidase (APX) activity, which degrades AsA, indicating that *MDHAR3* negatively regulates AsA accumulation. Transcriptomic analysis further revealed significant changes in genes related to ascorbate and aldarate metabolism. The study suggests that *MDHAR3* plays a complex role in AsA regulation, with potential species-specific functions in AsA metabolism. These findings offer valuable insights for enhancing fruit quality through genetic modification, particularly regarding antioxidant content and fruit health. Further research could investigate the interaction between MDHAR and APX activities to better understand their roles in regulating AsA levels in fruit [74].

Self-incompatibility (SI) is a crucial reproductive strategy in many flowering plants, preventing inbreeding and promoting genetic diversity [75,76]. However, in cultivated species, SI can significantly hinder fruit set and reduce agricultural yield [77]. Pitaya, a tropical fruit with increasing commercial importance, exhibits SI, which complicates pollination and fruit production [78,79]. The study by Wang et al. (2023) explores the molecular mechanisms behind SI in pitaya, focusing on the differences in gene expression between self-compatible and self-incompatible varieties. Using transcriptome sequencing, the researchers analyzed two varieties—“Jindu No. 1” (self-compatible) and “Cu Sha” (self-incompatible)—at various time points before and after pollination to capture changes in gene expression related to pollination. A total of 36,900 transcripts were identified, including 9167 novel transcripts, and 28,817 genes were annotated. The key findings revealed significant differences in DEGs in the stigmas, especially during the late post-pollination stage. The increased expression of protease genes was noted under cross-pollination. DEGs related to ribosomal, ubiquitination-mediated, and phyto-signaling pathways were highlighted, suggesting their role in regulating SI. The study also identified *HuS-RNase2* as a potential candidate gene involved in the pitaya SI system, providing a foundation for developing self-compatible varieties that could enhance crop yield and simplify cultivation [80].

Current research highlights the importance of understanding the genetic basis of SI in pitaya to breed self-compatible varieties and improve crop sustainability and productivity. Future research should focus on clarifying the roles of DEGs in SI mechanisms and their interaction with environmental factors. Developing genetic tools to manipulate SI traits will aid in creating self-compatible varieties, enhancing breeding strategies, and improving pitaya cultivation practices [19,80,81].

Advances in molecular research have significantly deepened our understanding of the genetic and biochemical mechanisms governing fruit development, quality traits, and stress responses. Through the application of genomics, transcriptomics, metabolomics, and gene editing, researchers are now better equipped to address challenges such as environmental stresses, disease susceptibility, and improving fruit quality. This comprehensive exploration of molecular tools and techniques highlights their transformative potential in optimizing fruit yield, enhancing nutritional value, and extending shelf life. As molecular technologies continue to evolve, they hold great promise for sustainable fruit production in the face of global challenges, ensuring food security and agricultural resilience. Future research integrating these cutting-edge approaches will drive innovation in breeding programs, ultimately benefiting both farmers and consumers alike.

The research presented in this Special Issue highlights the diverse and intricate molecular mechanisms that plants use to respond to environmental stresses and regulate key developmental processes. From enhancing cold tolerance through lipid desaturation in loquat to understanding the genetic control of flowering in pineapple, starch metabolism in banana, AsA regulation in tomato, and SI in pitaya, these studies collectively offer new insights that have the potential to revolutionize crop breeding and management practices. Looking forward, the application of molecular biology techniques such as transcriptomics, metabolomics, and gene editing will be crucial for advancing our understanding of plant stress tolerance and developmental regulation. These tools will help us develop crops that are more resilient to climate change, more productive, and of higher nutritional value. As we continue to explore the molecular foundations of these processes, the knowledge gained will be instrumental in meeting the global demand for sustainable agriculture.
